# Re-Audit of Adherence to Antipsychotic Prescribing Guidelines in a Psychiatric Unit for Older Adult Females: Evaluating Improvements and Compliance Post-Interventions

**DOI:** 10.1192/bjo.2025.10636

**Published:** 2025-06-20

**Authors:** Maria Moisan, Daisy Carroll, Ayebatonye Ajiteru

**Affiliations:** Kent and Medway NHS and Social Care Partnership Trust, Maidstone, United Kingdom

## Abstract

**Aims:** The aim of this audit was to assess adherence to antipsychotic prescribing guidelines in one of the Older Adults Female wards at Kent and Medway NHS and Social Care Partnership Trust (KMPT). Specifically, the audit focused on the documentation of key parameters for patients prescribed antipsychotics, including metabolic and cardiovascular risks, baseline assessments, and monitoring, in accordance with NICE guidelines. A re-audit was conducted in June–July 2024 to evaluate improvements following initial interventions. The original audit was published in BJPsych and presented at RCPsych 2024.

**Methods:** The initial audit in 2023 identified areas for improvement in documentation and monitoring of antipsychotic prescribing. Key parameters were assessed based on NICE guidelines. An action plan was developed and implemented, including refinements to the existing ward round process and the introduction of a physical health monitoring poster for junior doctors. A re-audit was conducted in 2024, analysing compliance with these parameters for two months of patient records. Compliance rates from 2023 and 2024 were compared to evaluate the effectiveness of the implemented changes.

**Results:** The re-audit showed significant improvements in several areas. In 2024, compliance for documenting patient age, diagnoses, and MHA status remained at 100%. The documentation of antipsychotic indication improved from 80% in 2023 to 100%. Consent to treatment and the MCA tab improved from 66.66% and 53.33% to 100%. Baseline ECG compliance rose from 86.66% to 100% and repeat ECGs within the recommended time frame increased from 53.33% to 79.17%. Blood tests showed significant improvements: fasting glucose/HbA1c (73.33% to 88%), lipid profile (73.33% to 92%), and liver function (73.33% to 100%). Repeat blood tests, such as fasting glucose, HbA1c, and lipid profile, also showed notable increases. Monitoring for side effects improved to 100%, compared with 46.66% in 2023. GP follow-up recommendations for physical health monitoring were fully documented in 2024, compared with 50% in 2023.

**Conclusion:** The re-audit demonstrated significant improvements in adherence to antipsychotic prescribing guidelines in one of the Older Adults Female wards at KMPT, particularly in areas related to baseline assessments, monitoring, and follow-up care, all in line with NICE guidelines. The changes made in response to the original audit, including refinements to the existing ward round process and the introduction of a physical health monitoring poster, while accommodating the rotation of junior doctors, were effective in enhancing documentation and compliance. Continued monitoring and future audits will be essential to sustain these improvements and further refine practice and documentation.

